# Insights Into the Seroprevalence, Clinical Spectrum, and Laboratory Features of Dengue and Chikungunya Mono-Infections vs. Co-infections During 2022–2023

**DOI:** 10.7759/cureus.92410

**Published:** 2025-09-15

**Authors:** Ishan Mewara, Deepti Chaurasia, Garima Kapoor, Nagaraj Perumal, Harendra Pratap Singh Bundela, Simmi Dube, Ankita Agarwal

**Affiliations:** 1 Department of Microbiology, Gandhi Medical College, Bhopal, IND; 2 State Virology Laboratory, Department of Microbiology, Gandhi Medical College, Bhopal, IND; 3 Department of General Medicine, Gandhi Medical College, Bhopal, IND

**Keywords:** arboviruses, central india, chikungunya, co-infection, dengue, seroprevalence

## Abstract

Background

Central India is hyperendemic for dengue and chikungunya virus infections, with yearly outbreaks being recorded. As these infections are clinically overlapping, co-infection of these viruses poses a diagnostic challenge. Therefore, it is imperative to identify them using serological or molecular tests. In this study, seroprevalence of dengue and chikungunya mono-infection as well as co-infections was estimated in suspected patients presented at our tertiary care center. Also, we compared the biochemical and other lab parameters of all the dengue and chikungunya mono-infected and co-infected IPD (Inpatient Department) patients.

Methods

A total of 3350 dengue and chikungunya suspected samples were recruited in this study. Sero-diagnosis of dengue was done using IgM MAC and NS‐1 antigen enzyme-linked immunosorbent assay (ELISA), and chikungunya by IgM MAC ELISA. Further, we compared the demographic characteristics, temporal variations, clinical features, and biochemical parameters in patients with dengue and chikungunya mono-infection vs. co-infection.

Results

Seropositivity for dengue (DENV) and chikungunya (CHIKV) mono-infections was observed to be 23.86% (n=800) and 13.30% (n=444), respectively, while seropositivity for dengue and chikungunya co-infections was observed to be 6.55% (n=220). Leucopenia was significantly higher among the co-infected patients as compared to DENV and CHIKV mono-infected cases. Hypokalemia was significantly higher among the DENV patients and co-infected patients as compared to the CHIKV patients. Urea and creatinine levels were significantly elevated among the CHIKV patients as compared to DENV and co-infected patients.

Conclusions

Leucopenia and hypokalemia were found to be significantly high among the co-infected patients. Clinically suspected samples should be tested for both viruses, especially in the monsoon and post-monsoon seasons, as high sero-prevalence has been observed. Simultaneous diagnosis of these infections is required to diagnose dual infections and thus triage patients for monitoring and initiating supportive treatment to prevent fatal complications.

## Introduction

Dengue and chikungunya are the most rapidly spreading mosquito-borne diseases caused by flavivirus and alphavirus, respectively. They have become a major public health concern in tropical and subtropical areas. Annually, an estimated 96 million clinical dengue infections occur worldwide. In India, these viruses are transmitted by *Aedes aegypti* and *Aedes albopictus* mosquitoes. In the absence of specific treatment, vector control measures form the mainstay of dengue control activities. The clinical presentations of both of these viral infections are similar, such as high-grade fever, chills, nausea, vomiting, headache, rash, arthralgia, and myalgia. As these infections show coinciding symptoms initially, it is difficult to distinguish them clinically. Dengue fever may progress to serious conditions like dengue hemorrhagic fever or dengue shock syndrome, whereas in chikungunya, debilitating arthralgia and joint pain up to several years have been observed in many patients [1,2].

The first ever case of dengue chikungunya co-infection was detected and reported from India in 1964 when chikungunya virus (CHIKV) and dengue type 2 virus (DENV-2) were isolated from a single blood specimen of a patient at Vellore, South India [3]. Since then, various cases of co-infection have been reported. In a study reported by Chahar et al., CHIKV nucleic acids were detected from 17 clinically suspected cases of DENV/CHIKV co-infection during the dengue fever outbreak of 2006 in Delhi, out of which six were co-infected with DENV [4]. Subsequently, in 2011, molecular and serological tests specific for both DENV and CHIKV infections were performed on 87 acute-phase blood samples collected from suspected patients in Delhi, wherein co-infection with DENV and CHIKV was observed in 10% of the samples [5]. During the 2015 Delhi dengue outbreak, co-infection of DENV and CHIKV was observed in 12 patients among the 334 patients tested for both infections [6]. In another study conducted in New Delhi during 2016, CHIKV and DENV were reported in five (9%) samples among the 55 samples tested for both the viruses [7].

A study conducted in 2017 to understand the DENV-CHIKV infection dynamics during West Bengal outbreaks revealed that dengue endemic areas overlap with chikungunya-affected areas, and both the viruses were transmitted by the same vector, *Aedes aegypti* [8]. Blood was collected from 326 symptomatic febrile patients, where co-infection was found in 23% patients [8]. Another study conducted in the northwestern region of Punjab reported that out of the total 3160 samples collected from suspected patients for dengue infection, 283 samples were tested for both viruses, of which 27 sera were co-positive (9.54%) [9].

In clinical practice, the comparison of mono-infections vs. co-infections plays a role in informing disease severity, treatment, and improving prognosis. In public health practice, it is important to understand the disease transmission trends and predict the severity and patterns of outbreaks in order to develop more effective prevention and control strategies. Despite the recent surge in DENV and CHIKV cases, there is limited data on seroprevalence, clinical and laboratory parameters of mono-infected vs. co-infected cases. In view of this, we conducted this study with the following objectives: (a) Estimating the seroprevalence of dengue and chikungunya mono-infection as well as co-infections in suspected patients presented at our tertiary care center; (b) Comparison of the biochemical and other lab parameters of all the dengue and chikungunya mono-infected and co-infected inpatient department (IPD) patients.

## Materials and methods

Study design and study ethics

This hospital-based cross-sectional study was conducted at the State Virology Laboratory, Department of Microbiology, Gandhi Medical College, Bhopal, Madhya Pradesh, over a 24-month period (January 2022 to December 2023). Patients suspected of having dengue and/or chikungunya, presenting to the Medicine and Pediatrics Outpatient and Inpatient Departments of our tertiary care teaching hospital, who met the inclusion criteria were recruited in the study. A person is considered a suspect for dengue/chikungunya if they have a sudden high fever along with any of the two other symptoms, like headache, chills, muscle pain, joint pain, nausea, and rash [1].

The study protocol was approved (Approval No: 32092/MC/IEC/2022) by the Institutional Ethics Committee, Gandhi Medical College, Bhopal. Written informed consent was obtained from adult patients, while parents/guardians provided assent on behalf of minors.

Inclusion and exclusion criteria

All patients suspected of having dengue/chikungunya viruses were included in the study. The following exclusion criteria were adopted: (a) Hemolyzed, inadequate, or leaked sample; (b) Samples without requisition form or requisition form without sample; (c) Incomplete or illegible form, especially regarding date of onset of symptoms, date of sample collection, and clinical details.

Blood collection

Blood samples were collected in Vacutainer (Becton, Dickinson and Company, Franklin Lakes, NJ, USA) vials during the first visit to the hospital. Clinical and demographic information was meticulously recorded using a standardized case-record form. The collected blood samples were allowed to clot at ambient temperature (20-25°C). Subsequently, samples were centrifuged at 3000 rpm for 3 min to facilitate serum separation. The isolated serum was then transferred to microcentrifuge tubes for serological testing and stored at 2-8°C until analysis. For long-term storage, serum samples are stored at -80°C.

Serological analysis

Serological diagnosis of dengue and chikungunya infections was performed using the following kits: Dengue NS1 Ag enzyme-linked immunosorbent assay (ELISA) kit (Medsource Ozone Biomedicals Pvt. Ltd., India), Dengue IgM Capture ELISA kit (National Institute of Virology, Pune, India), and Chikungunya IgM Capture ELISA kit (National Institute of Virology, Pune, India).

The Dengue NS1 Ag ELISA procedure is based on the principle of sandwich ELISA. Briefly, a diluted patient sample was added to the microwells coated with monoclonal anti-dengue NS1 antibodies and incubated. Following this, monoclonal anti-dengue NS1 conjugated to horseradish peroxidase (HRP) enzyme was added. After this, washing was done to remove the unbound components. Bound enzyme was detected by adding a substrate. The reaction was stopped after 15 min, and absorbance was recorded at 450 nm. The sensitivity and specificity of the dengue NS1 Ag ELISA kit are 100% and 98%, respectively.

In the dengue/chikungunya IgM capture ELISA, a diluted patient sample was added to the microwells coated with anti-human IgM to capture IgM antibodies present in the patient’s serum. Following this, the dengue/chikungunya antigen was added for binding with the captured human IgM in the sample. Unbound antigen was removed by washing. After this, biotinylated anti-Den/Chik monoclonal antibodies were added, followed by the addition of Avidin-HRP. Subsequently, chromogenic substrate was added, and the reaction was stopped, and absorbance was recorded at 450 nm. The sensitivity and specificity of the dengue IgM Capture ELISA kit are 98.53% and 98.84%, respectively. The sensitivity and specificity of the Chikungunya IgM Capture ELISA kit are 95% and 98%, respectively.

Statistical analysis

For quantitative variables, descriptive statistics (mean±standard deviation and percentages) were calculated. A comparison of clinical characteristics in patients with dengue and chikungunya mono-infection vs. co-infection was done as per the history mentioned in their respective case record forms (CRFs). A comparison of biochemical and other lab parameters of all the dengue and chikungunya mono-infected and co-infected IPD patients during the study period was done using the chi-square test. Statistical analysis was performed on GraphPad Prism 10.0 software (Dotmatics, Boston, MA, USA). Statistical significance was set at p<0.05 and p<0.01.

## Results

Seropositivity in mono-infections vs. co-infection

Out of 3350 patients suspected of having dengue/chikungunya, seropositivity for dengue and chikungunya mono-infections was observed to be 23.86% and 13.30% of the patients, respectively, while seropositivity for dengue and chikungunya co-infections was observed in 6.55% of the patients.

Comparison of demographic characteristics (age and gender) in patients with dengue and chikungunya mono-infection vs. co-infection

A comparison of age in patients with dengue and chikungunya mono-infection vs co-infection revealed the most common age group affected for dengue was 15-30 years with 1527 samples received, out of which dengue seropositive were 414 samples (27.11%), while the most affected age group for chikungunya and co-infected was 46-60 years with 311 samples received, out of which chikungunya sero-positive and co-infected were 55 (17.68%) and 31 (9.96%) samples respectively (Table 1).

**Table 1:** Comparison of demographic characteristics (age) in patients with dengue and chikungunya mono-infection vs. co-infection.

Age (years)	Total number of suspected patients	Dengue seropositive patients, n (%)	Chikungunya seropositive patients, n (%)	Co-positive patients, n (%)
0-5	328	46 (14.00%)	23 (7.01%)	8 (2.43%)
6-14	446	91 (20.40%)	53 (11.88%)	26 (5.82%)
15-30	1527	414 (27.11%)	204 (13.35%)	112 (7.33%)
31-45	571	142 (24.86%)	86 (15.06%)	34 (5.95%)
46-60	311	76 (24.43%)	55 (17.68%)	31 (9.96%)
>60	167	31 (18.56%)	23 (13.77%)	9 (5.38%)
Total	3350	800 (23.86%)	444 (13.30%)	220 (6.55%)

Of the 3350 patients, 1808 were men and 1542 were women. Out of 1808 men, 488 patients (14.56%) were seropositive for dengue infection, 217 patients (12%) were seropositive for chikungunya infection, and 108 patients (5.97%) were serologically co-positive for dengue chikungunya co-infection. Also, out of 1542 female patients, 308 (19.97%) were seropositive for dengue infection, 216 (14%) were seropositive for chikungunya infection, and 108 (7%) were serologically co-positive for dengue-chikungunya co-infection.

Comparison of temporal characteristics in patients with dengue and chikungunya mono-infection vs co-infection

**Figure 1:**
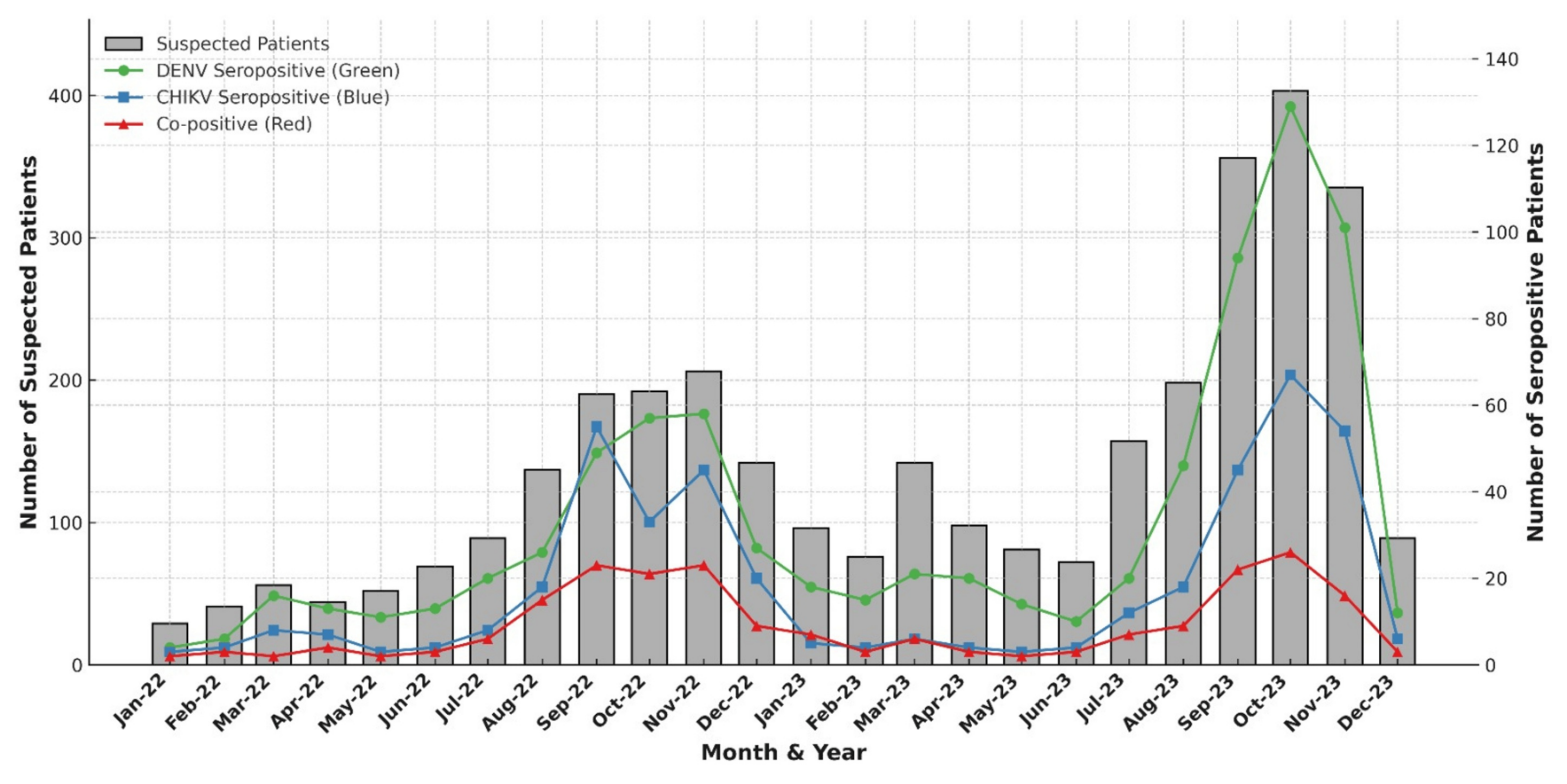
Temporal trends in suspected (n=3350) and confirmed dengue, chikungunya, and co-infection cases, central India (2022–2023). Monthly distribution of suspected cases and laboratory-confirmed seropositive cases of dengue virus (DENV), chikungunya virus (CHIKV), and co-infections shows that the maximum number of samples was received during November 2022 and October 2023. The peak seropositivity for these infections was observed during the post-monsoon months of September, October, and November in both years. Suspected cases are represented by grey bars (left Y-axis), while DENV (green circles), CHIKV (blue squares), and co-positive cases (red triangles) are represented by coloured lines (right Y-axis).

The study was conducted from January 2022 to December 2023. Out of 3350 dengue and chikungunya suspected samples tested, the majority of the samples were received during November 2022 and October 2023. The peak seropositivity for dengue chikungunya mono-infection and co-infection was observed during the post-monsoon months of September, October, and November each year (Figure 1).

Comparison of clinical characteristics in patients with dengue and chikungunya mono-infection vs. co-infection

Of the 3350 dengue chikungunya suspected samples, all the 3350 patients (100%) had fever as the most common symptom. The other most prominent symptom was chills, which was observed in 136 (17.19%) dengue-seropositive patients, 71 (16.16%) chikungunya-seropositive patients, and 37 (17.17%) co-infected patients. Fever followed by chills, myalgia, pain in the abdomen, vomiting, and headache were the most prominent clinical features in that order. Other minor clinical features observed were rigors, malaise, cough, sore throat, diarrhea, retro-orbital pain, rhinorrhea, rash, dark urine, etc. (Table 2).

**Table 2:** Comparison of clinical characteristics in patients with dengue and chikungunya mono-infection vs. co-infection.

Clinical signs and symptoms	Dengue seropositive patients, n (%)	Chikungunya seropositive patients, n (%)	Co-positive patients, n (%)
Fever	800 (100%)	444 (100%)	220 (100%)
Chills	136 (17.19%)	71 (16.16%)	37 (17.17%)
Rigors	37 (4.71%)	36 (8.20%)	12 (5.55%)
Malaise	43 (5.40%)	35 (7.96%)	10 (4.54%)
Myalgia	88 (11.09%)	62 (13.93%)	32 (14.64%)
Rash	1 (0.13%)	0 (0%)	0 (0%)
Vomiting	86 (10.81%)	48 (10.94%)	20 (9.09%)
Headache	63 (7.90%)	35 (7.96%)	20 (9.09%)
Arthralgia	39 (4.85%)	22 (4.97%)	13 (6.06%)
Retro-orbital pain	20 (2.49%)	12 (2.73%)	3 (1.51%)
Jaundice	15 (1.94%)	11 (2.48%)	2 (1.01%)
Pain in abdomen	110 (13.73%)	56 (12.68%)	30 (13.63%)
Cough	40 (4.99%)	25 (5.72%)	19 (8.58%)
Sore throat	39 (4.85%)	26 (5.97%)	15 (7.07%)
Rhinorrhea	1 (0.13%)	3 (0.74%)	1 (0.50%)
Diarrhea	28 (3.60%)	16 (3.73%)	5 (2.52%)
Dark urine	7 (0.97%)	4 (0.99%)	1 (0.50%)

Comparison of biochemical and other laboratory parameters in patients with dengue and chikungunya mono-infection vs. co-infection

Out of 1464 seropositive patients, the seriously ill (n=409) were admitted to the hospital. Of these 409 IPD patients, 276 were dengue seropositive, 42 were chikungunya seropositive, and 91 patients were serologically co-infected. The biochemical parameters of these patients admitted during the study period were compared. Anemia was found in 45.2% dengue patients, 54.7% chikungunya patients, and in 44.4% patients with co-infections. No significant difference was observed in anemia among the three groups (p>0.05). Leucopenia was found in 22.8% dengue patients, 26.2% chikungunya patients, and in 46.1% patients with co-infections. Leucopenia observed in our study was significantly higher among the co-infected patients as compared to DENV mono-positive patients (p<0.01) and CHIKV mono-positive patients (p<0.05). Thrombocytopenia was reported in 62.3% patients with dengue infection, 54.7% patients with chikungunya infection, and 71.4% patients with co-infections. No significant difference was observed in comparing thrombocytopenia among the three groups (p>0.05). Hyponatremia was reported in 57.2% patients with dengue infection, 59.5% patients with chikungunya infection, and 58.2% patients with co-infections. No significant difference was observed in comparing hyponatremia among the three groups in our study (p>0.05). Hypokalemia was reported in 21% patients with dengue infection, 4.7% patients with chikungunya infection, and 27.5% patients with co-infections. Hypokalemia observed in our study was significantly higher among the DENV mono-positive and co-infected patients as compared to CHIKV mono-infected patients (p<0.01). Elevated urea levels were reported in 22.1% patients with dengue infection, 42.8% patients with chikungunya infection, and 24.2% patients with co-infections. Urea levels were significantly elevated among the CHIKV mono-positives as compared to DENV mono-infected and co-infected patients (p<0.01, p<0.05). Elevated creatinine levels were reported in 21% patients with dengue infection, 52.4% patients with chikungunya infection, and 31.8% patients with co-infections. Creatinine levels were significantly elevated among the CHIKV mono-positive patients as compared to DENV mono-infected patients and co-infected patients (p<0.01, p<0.05). Liver parameters were deranged in our study; serum glutamic pyruvate transaminase (SGPT) was deranged in 44.2%, 52.4% and 49.4% patients with dengue, chikungunya, and co-infection, respectively. Serum glutamic-oxaloacetic transaminase (SGOT) was elevated in 54.3%, 52.4% and 61.5% patients with dengue, chikungunya, and co-infection, respectively. Also, hypoalbuminemia was reported in 63%, 71.4% and 59.3% patients with dengue, chikungunya, and co-infection, respectively. No significant difference was observed in liver enzyme derangement among the three groups (p>0.05) (Table 3).

**Table 3:** Comparison of biochemical and other laboratory parameters among IPD patients (n=409) with dengue and chikungunya mono-infection vs co-infection. A test of significance of differences between two independent proportions was performed using chi chi-square test. p value<0.05, p<0.01 is considered significant. CHKV: chikungunya virus; DENV: dengue virus; SGPT (ALT): serum glutamic pyruvate transaminase (alanine aminotransferase); SGOT (AST): serum glutamic-oxaloacetic transaminase (aspartate aminotransferase); IPD: inpatient department.

Parameters	Normal range	DENV n=276 (%) (A)	CHIKV n=42 (%) (B)	DENV + CHIKV co-infection n=91 (%) (C)	p-value		
					Group A and B	Group A and C	Group B and C
Anemia	11-16 g/dL	125 (45.29)	23 (54.76)	40 (44.44)	0.25	0.82	0.25
Leukopenia	4-11×10^3^/mm^3^	63 (22.82)	11 (26.19)	42 (46.15)	0.63	p<0.01	p<0.05
Thrombocytopenia	150-400×10^3^/mm^3^	172 (62.31)	23 (54.76)	65 (71.42)	0.35	0.11	0.06
Hyponatremia	135-145 mEq/L	158 (57.24)	25 (59.52)	53 (58.24)	0.79	0.87	0.89
Hypokalemia	3.5-5.5 mEq/L	60 (21.05)	2 (4.76)	25 (27.47)	p<0.01	0.26	p<0.01
Elevated urea	15-40 mg/dL	61 (22.10)	18 (42.85)	22 (24.17)	p<0.01	0.69	p<0.05
Elevated creatinine	0.5-1.0 mg/dL	58 (21.01)	22 (52.38)	29 (31.86)	p<0.01	p<0.05	p<0.05
Elevated SGPT (ALT)	5-35 U/L	122 (44.20)	22 (52.38)	45 (49.45)	0.32	0.39	0.75
Elevated SGOT (AST)	5-37 U/L	150 (54.34)	22 (52.38)	56 (61.53)	0.81	0.23	0.32
Hypoalbuminemia	3.5-5.0 g/dL	174 (63.04)	30 (71.42)	54 (59.34)	0.30	0.53	0.18

## Discussion

In this study, we have assessed the seroprevalence of dengue and chikungunya mono-infection as well as co-infections among suspected patients who presented at our tertiary care center during the year 2022-2023. Out of the 3350 dengue and/or chikungunya suspected samples received during the study period, we have observed 23.86% seropositivity for dengue, 13.30% seropositivity for chikungunya and 6.55% seropositivity for dengue-chikungunya co-infections.

Demographic characteristics

A comparison of demographic characteristics (age) in patients with dengue and chikungunya mono-infection vs. co-infection revealed that most of the dengue seropositive patients were found in the age group of 15-30 years. However, the maximum number of chikungunya sero-positive and co-infected patients was found in the age group 46-60 years. A comparison of infection on the basis of gender revealed that men were affected more than women by dengue infection. However, chikungunya mono-infection showed a slightly higher affinity towards female patients in our study. The findings are in accordance with a study conducted in Ahmedabad, where chikungunya prevalence was higher in women [10].

Temporal variations

Temporal variations studied in patients with dengue and chikungunya mono-infection vs. co-infection revealed that the months of September, October, and November had the highest number of samples with maximum seropositivity. These findings are in accordance with a previous study conducted in central India, which also demonstrated the increased incidence of dengue during these months [11]. This mandates the need for stringent preventive measures for arthropod-borne viral illness in central India before the initiation of the monsoon period.

Clinical features

Among all the dengue, chikungunya mono-infected and co-infected patients, the most common clinical presentation was fever, which was present in all the patients. The other common features involved in all three patient groups were chills, pain in the abdomen, myalgia, nausea, vomiting, headache, and malaise, while rhinorrhea, bone and joint pain, retro-orbital pain, rash, jaundice, diarrhea, and dark urine were observed in some patients only. Patients with chikungunya mono-infection reported myalgia, rigors, and malaise more frequently as compared to those with dengue mono-infection. In terms of clinical symptoms, no significant difference was observed among the mono-infected and co-infected patients.

Biochemical and other laboratory parameters

Further, we compared the biochemical and other laboratory parameters of the admitted patients. No significant difference was observed in anemia prevalence among the three groups (p>0.05). Leucopenia observed in our study was significantly higher among the co-infected patients as compared to DENV mono-positive patients (p<0.01) and CHIKV mono-positive patients (p<0.05). Several studies have reported leucopenia in dengue [12,13]. Thrombocytopenia has been reported to be more common in dengue as compared to CHIKV mono-infection [9]. Another study reported thrombocytopenia in all the co-infected patients as compared with mono-infections [13]. Similarly, it is found in a high number of co-infected patients in our study; however, no significant difference was observed among the three groups (p>0.05).

Hyponatremia and hypokalemia are the most common electrolyte disturbances in dengue fever. The levels of serum sodium play a significant role in the prognosis of dengue fever and dengue-associated complications [14]. A high number of IPD patients (>55%) infected with DENV or CHIKV or both had hyponatremia. However, the difference was not significant (p>0.05). Hypokalemia is also reported in dengue patients [15]. In our study, it was significantly higher among the DENV mono-positive and co-infected patients as compared to CHIKV mono-infected patients (p<0.01). High creatinine indicates severe renal impairment. It is known that CHIKV can replicate in muscular cells, causing muscle cell destruction, formation of creatinine by the degradation of muscular creatinine, and increasing its levels in the blood [16]. Therefore, urea and creatinine levels were significantly elevated among the CHIKV mono-positives as compared to DENV mono-infected (p<0.01) and co-infected patients (p<0.05). The liver is a frequently involved organ in dengue infection [17]. Mild to moderate elevation of transaminases in both dengue fever and dengue hemorrhagic fever has been reported [18]. Liver parameters (SGPT, SGOT, albumin) were also elevated in our study; however, a significant difference was not observed among the three groups (p>0.05).

Comparison with other co-infection studies

Several studies on dengue and chikungunya co-infection have been conducted throughout India. A recent study conducted in Uttarakhand by Badoni et al. reported 1.4% seropositive co-infections out of 279 samples [19]. However, molecular confirmation of the viral genome was not done in their study. Many studies have been reported from Delhi on dengue chikungunya co-infection. Different studies have reported coinfection in Delhi at different times, for instance, 8.69% co-infection observed among 69 samples [4], 10% seropositivity with dengue and chikungunya co-infections [5], 3.59% dengue-chikungunya co-infection observed among 334 samples [6], and 9% co-infection observed among 55 samples tested for both DENV and CHIKV [7]. A cross-sectional analysis in West Bengal demonstrated 16% dengue-chikungunya co-infection among 326 suspected samples [8]. A study conducted in northwestern Punjab demonstrated that 283 samples were tested for both viruses, out of which 9.54% of samples were positive for DENV and CHIKV co-infection [9]. In a study conducted in central India, out of 138 samples screened for dengue, 15.2% were positive for DENV, and out of 119 samples screened for chikungunya, 10.9% were positive for CHIKV [20]. Different co-infection studies from India have been tabulated in Table 4.

**Table 4:** Summary of studies conducted for dengue chikungunya co-infections from India. ELISA: enzyme-linked immunosorbent assay; PCR: polymerase chain reaction.

S. No.	Citation	Place	Study design	Sample size	Positive for co-infection	Co-infection (%)	Age	Diagnostic test
1	Chahar et al. (2009) [4]	Delhi	Cross-sectional	69	6	9	All ages	PCR
2	Kalawat et al. (2011) [21]	Tirupati (Andhra Pradesh)	Retrospective analysis	72	2	3	All ages	IgM ELISA
3	Taraphdar et al. (2012) [22]	West Bengal	Cross-sectional	550	68	12	All ages	IgM ELISA, PCR
4	Afreen et al. (2014) [5]	Delhi	Cross-sectional	87	9	10	All ages	IgM ELISA, PCR
5	Saswat et al. (2015) [23]	Khurda (Odisha), Aurangabad (Maharashtra)	Cross-sectional	222	43	19	All ages	IgM ELISA, PCR
6	Shaikh et al. (2015) [24]	Karnataka	Cross-sectional	6554	532	8	NM	IgM ELISA
7	Galate et al. (2016) [25]	Mumbai (Maharashtra)	Cross-sectional	200	19	9.5	13–60	IgM ELISA
8	Londhey et al. (2016) [26]	Mumbai (Maharashtra)	Prospective observational study	300	30	10	All ages	IgM ELISA, PCR
9	Morch et al. (2017) [27]	Assam, Bihar, Chhattisgarh, Maharashtra, Andhra Pradesh, Tamil Nadu	Cross-sectional	98	25	26	34 (mean)	IgM ELISA
10	Mukherjee et al. (2017) [8]	Kolkata (West Bengal)	Cross-sectional	326	53	16	All ages	IgM ELISA, PCR
11	Savargaonkar et al. (2018) [6]	Delhi	Cross-sectional	334	12	3.59	All ages	IgM ELISA, PCR
12	Hisamuddin et al. (2018) [7]	Delhi	Cross-sectional	55	5	9	All ages	IgM ELISA, PCR
13	Kaur et al. (2018) [9]	Punjab	Cross-sectional	283	27	9.54	All ages	IgM ELISA
14	Gupta et al. (2020) [28]	Mumbai (Maharashtra)	Prospective	200	9	4.5	All ages	IgM ELISA, PCR
15	Badoni et al. (2023) [19]	Uttarakhand	Cross-sectional	279	4	1.43	All ages	IgM ELISA
16	Sreedevi et al. (2023) [29]	Warangal (Telangana)	Cross-sectional	10339	153	7.8	All ages	IgM ELISA
17	Gopinath et al. (2023) [30]	Tamil Nadu	Retrospective	3753	103	2.7	All ages	IgM ELISA

It has been suggested that the ELISA can be used in those centers where molecular testing cannot be performed and will help to detect antibodies even after the viraemic stage. Since molecular testing is required to confirm DENV or CHIKV infection in the early stages of the infection, the sensitivity of molecular methods decreases in the advanced stages of the infection due to the initiation of a rapid immune response, which is followed by a reduction in viral load. Thus, at this point, IgM ELISA and other serological investigations are more sensitive and reliable. Hence, it is advocated that reverse transcriptase polymerase chain reaction (RT-PCR) is not recommended beyond the febrile stage of infection [19].

Another study has observed co-circulation of DENV and CHIKV in central India [20]. The clinical features and laboratory parameters of only dengue and chikungunya mono-infection were described, and no co-infection was reported. Since no comprehensive data is available to compare and discuss dengue-chikungunya co-infections prevailing in central India, our study is the first to report a 6.5% DENV-CHIKV co-infection out of the 3350 suspected samples. Thus, our study will provide distinct features for the prevailing DENV CHIKV co-infections in central India. This underscores the need for targeted prevention strategies and improved diagnostic methods.

Study limitations

Firstly, it was a cross-sectional study conducted at a single center and, therefore, might have been subjected to selection bias. Secondly, the absence of adjusted analysis might have led to the study findings being influenced by confounding variables that were not taken into account. Thirdly, a molecular assay was not performed due to limited resources; therefore, there might be a possibility of potential cross-reactivity among arboviral IgM assays. Fourthly, we could not perform a subsequent follow-up of patients for the outcome. Lastly, the information pertaining to laboratory parameters could be collected only from IPD patients. We understand that these issues impact results; however, future studies can address these gaps.

## Conclusions

Clinically suspected samples should be tested for both viruses, especially in the monsoon and post-monsoon seasons, as high sero-prevalence has been observed. As leukopenia and hypokalemia have been detected among a high number of co-infected patients, these parameters should be evaluated as they could trigger suspicion of co-infection. This study provides clinicians with detailed insights into the clinical and laboratory differences between dengue, chikungunya, and their co-infections, enabling more accurate diagnosis and targeted patient management. This will not only benefit clinicians, but also public health officials or policymakers for the implementation of effective vector surveillance and control measures to significantly alleviate the vector populations in this area.
